# The relationship between red blood cell distribution width and islet β-cell function indexes in patients with latent autoimmune diabetes in adults

**DOI:** 10.1186/s12902-023-01435-x

**Published:** 2023-08-24

**Authors:** Xiuli Fu, Qin Tan, Wei Wei, Sheng Ding, Zhongjing Wang

**Affiliations:** 1Department of Endocrinology, The Central Hospital of Wuhan, Tongji Medical College, Huazhong University of Science and Technology, Wuhan, 430014 China

**Keywords:** Latent autoimmune diabetes in adults, Red blood cell distribution width, β-cell function, Glycosylated hemoglobin A1c

## Abstract

**Aims:**

The objective of this study is to explore the relationship between red blood cell distribution and islet β-cell function indexes in patients with Latent Autoimmune Diabetes in Adults.

**Methods:**

A total of 487 LADA patients were enrolled in this cross-sectional study. Patients were divided into three groups according to RDW tertiles. Clinical and laboratory measurements of age, height, weight, duration of diabetes, blood pressure, RDW, glycosylated hemoglobin A1c (HbA1c), C-peptide and blood lipids were performed. Homeostasis model assessment of insulin resistance (HOMA-IR) and homeostasis model assessment of β-cell function (HOMA-β) were assessed using homeostasis model assessment (HOMA) based on fasting blood glucose (FBG) and fasting C-peptide index (FCP). Correlations and multiple linear regressions were implemented to determine the association of RDW and islet function indexes.

**Results:**

As the increase of serum RDW level, the presence of β-cell secretion increased(P < 0.05). Correlation analysis indicated that there were significant correlations between RDW and male sex, age, duration, TG, Cr, FCP, and HOMA-β in all subjects. Multiple linear regressions indicated that RDW was significantly correlated with HOMA-β in the total population in both unadjusted and adjusted analysis. This finding could be reproduced in the subgroup of low GAD titers for HOMA-β. RDW were significantly associated with HbA1c in LADA patients with high GAD titers, but the correlation was not found in subgroup with low GAD titers in either unadjusted analyses or adjusted analysis.

**Conclusions:**

RDW is associated with β-cell function assessed by HOMA-β after adjusting for covariates in LADA patients with low GAD titers.

## Background

Red Blood Cell Distribution Width (RDW) is an index of red blood cell (RBC) size variability and is calculated as the standard deviation in RBC size divided by the mean corpuscular volume (MCV). Emerging evidence suggests that, besides RBC abnormalities, diverse human pathologies have been frequently associated with anisocytosis. RDW is also considered as a novel inflammation marker associated with poor prognosis in many clinical scenarios and populations, including patients with increased mortality risk with COVID-19, acute stroke, myocardial infarction, and type 2 diabetes [1–3].

Diabetes mellitus is a global concern that causes an enormous burden to society and individuals. Latent autoimmune diabetes in adults (LADA) is a kind of adult-onset autoimmune diabetes, which shows the feathers of both type 1 and type 2 diabetes mellitus[4]. It is a heterogeneous form of diabetes with a pathogenesis that includes both autoimmune destruction of pancreatic beta cells as well as some degrees of insulin resistance [5]. These genotypes and its interaction with environmental factors result in a wide range of clinical phenotypes. However, the clinical characterization of these patients in the primary care setting is lacking, and its clinical progression is unpredictable. Despite its important clinical relevance, Patients with LADA are often misdiagnosed and show a sustained worse glycemic control compared to T2DM.

Studies pointed that an increase in RDW level has been shown to be closely related to T2DM or its complications and a marker of worsening diabetes control [6–8]. A reduction in counts of red blood cells and levels of haemoglobin in Swedish TEDDY children with multiple islet autoantibodies has been shown, and RDW values are significantly associated with HOMA-β and HbA1c[9].However, there are currently little clinical research on the correlation between red blood cell distribution and islet β-cell function among patients with LADA. Considering the importance of patients with LADA, the purpose of this study was to investigate the relationship between RDW and islet β-cell function in patients with LADA.

## Methods

### Subjects

The study was a retrospective, observational study that analyzed the data gathered previously. We enrolled 487 patients with diagnosed LADA at The Central Hospital of Wuhan from January 2018 to December 2021. The inclusion criteria for LADA patients were as follows:(1) diagnosed with diabetes (1999 World Health Organization [WHO] diagnostic criteria) ; (2) no ketoacidosis in the first 6 months after diagnosis of diabetes; (3) insulin independence(insulin use < 1 month) for 6 months after diagnosis; (4)GAD autoantibody(GADA) positive; (5) fasting C peptide(FCP) > 0.2ng/ml. Patients meeting any one of the following criteria should be excluded from the study:(1) subjects with secondary diabetes, pregnancy or a malignancy; (2) subjects in the state of ketoacidosis; (3) subjects who had used immunosuppressive drugs, antibiotics or steroid medication in the past 3 months; (4) other severe diseases; (5) lack of necessary laboratory or physical examination data.

For the included patients, clinical data were extracted from the electronic medical records, including age, sex, duration of T2DM, height, weight, hypertension, systolic blood pressure (SBP), diastolic blood pressure (DBP), complete blood count, lipid profile, glucose metabolism indexes and use of insulin. Body mass index (BMI) was calculated as the weight in kilograms divided by the square of the height in meters.

Blood samples were collected from the patients after 8 h of fasting. Biomedical measurements were tested for serum or plasma separation upon blood collection. Overnight fasting blood samples were collected from each participant to test for fasting blood glucose (FBG), serum total cholesterol (TC), low-density lipoprotein cholesterol (LDL), high-density lipoprotein cholesterol (HDL), triglycerides (TG), RDW, serum uric acid (SUA), glycosylated hemoglobin A1c (HbA1c) and liver/renal functions. After fasting blood samples were collected, the LADA patients took a bread meal test, which is a steamed bread (100 g flour), and then blood samples were collected 2 h after the meal to measure the postprandial C-peptide. HOMA-β and HOMA-IR were calculated from fasting C-peptide and glucose using the HOMA calculator [10].

### Statistical analysis

All analyses were performed using IBM Statistical Product and Service Solutions (SPSS) statistics 26.0 software. Clinical characteristics were described in all of the participants using mean and standard deviations (SD) for continuous variables and percentages for categorical variables. The normally distributed data were analyzed using the student’s t test or the one-way analysis of variance (ANOVA) with Bonferroni corrections for post hoc analysis. The non-normally distributed data were analyzed using the Mann-Whitney test or the Kruskal-Wallis H test to identify statistical differences between groups. Categorical variables described the number and percentage of each type, and comparisons between groups were processed by chi-square(χ^2^) test or Fisher’s exact test. We evaluated the association between clinical biological markers and RDW using Pearson’s correlation for parametric variables and Spearman’s rank correlation for nonparametric variables. Multiple linear regression analysis was conducted to determine whether RDW was associated with HOMA-β, HOMA-IR and HbA1c with or without adjusting for potential confounding factors. In all statistical comparisons, *P* < 0.05 was considered statistically significant.

## Results

A total of 487 individuals participated in this study in which men accounted for 47.2% and women accounted for 52.8%. The patients were categorized into three groups based on RDW tertiles. Table 1 showed the differences of clinical and biochemical characteristics among groups. In our study, there were significant differences in gender, age, duration of diabetes, TG, Cr, FCP and HOMA-β among groups(P < 0.05), while no significant differences were found in BMI, SBP, DBP, TC, LDL, HDL, ALT, AST, SUA, HbA1c, and HOMA-IR. There was a gradually increased trend of FCP across the three groups. The Kruskal-Wallis H test showed significant difference between tertile 1 and tertile 3 of HOMA-β values and use of insulin, but no statistical significance was demonstrated between tertile 1 and tertile 2 or between tertile 2 and tertile 3.

**Table 1:** Clinical characteristics and islet function indexes of total subjects according to RDW tertiles

	I(11.2–12.4%)	II(12.5–12.9%)	III(13.0–18%)	P
n	163	164	160	
male (%)	93(57.1%)^#Δ^	75(45.7%)^*Δ^	62(38.8%)^*#^	< 0.01
Age (years)	57.0 ± 16.9^#Δ^	58.4 ± 16.7^*Δ^	60.1 ± 17.1^*#^	< 0.01
duration(years)	6.3 ± 4.5^#Δ^	6.9 ± 4.6^*Δ^	7.1 ± 4.8^*#^	< 0.01
BMI (kg/m^2^)	21.8 ± 4.2	22.7 ± 4.6	22.6 ± 4.3	0.546
SBP (mmHg)	127.9 ± 18.8	129.3 ± 18.4	128.8 ± 18.9	0.509
DBP (mmHg)	71.7 ± 18.2	71.3 ± 18.6	72.1 ± 17.8	0.648
TG (mmol/L)	1.6 ± 0.5^#Δ^	1.5 ± 0.6^*Δ^	1.4 ± 0.6^*#^	0.043
TC (mmol/L)	4.3 ± 0.8	4.4 ± 0.7	4.4 ± 0.9	0.160
LDL (mmol/L)	2.6 ± 0.7	2.7 ± 0.6	2.8 ± 0.7	0.314
HDL (mmol/L)	1.1 ± 0.4	1.0 ± 0.4	1.0 ± 0.4	0.087
GAD(IU/ml)	119.3 ± 64.8	116.0 ± 63.3	116.2 ± 65.2	0.569
ALT (U/L)	19.2 ± 13.5	21.6 ± 13.4	21.5 ± 13.9	0.645
AST (U/L)	19.4 ± 12.4	21.7 ± 13.7	21.1 ± 12.9	0.626
Cr (umol/L)	61.5 ± 30.7^#Δ^	63.6 ± 30.2^*Δ^	66.6 ± 30.7^*#^	0.021
SUA (umol/L)	318.9 ± 147.7	330.6 ± 157.9	322.2 ± 155.3	0.542
HbA1c(%)	9.2 ± 2.3	9.1 ± 2.4	9.0 ± 2.5	0.057
FCP (ng/ml)	1.4 ± 0.7^#Δ^	1.5 ± 0.7^*Δ^	1.6 ± 0.7^*#^	0.037
P2hCP (ng/ml)	1.8 ± 0.7	2.2 ± 0.8	2.5 ± 0.8	0.068
Use of insulin(%)	76(46.6%)^Δ^	75(45.7%)	73(45.6%)^*^	0.229
HOMA-IR	1.6 ± 0.6	1.6 ± 0.6	1.6 ± 0.6	0.468
HOMA-β	12.0 ± 6.3^Δ^	13.8 ± 6.1	15.2 ± 6.4^*^	0.032

Values are mean ± SD,*P < 0.05 VS tertile 1; # P < 0.05 VS tertile 2;ΔP < 0.05 VS tertile 3

Patients were divided into two groups based on the GAD values: LADA-1 group (GAD ≥ 180 IU/ml)) and LADA-2 group (GAD < 180 IU/ml)). The results for comparison between groups in demographic data and laboratory measurements were shown in Table 2. The levels of BMI, TG, LDL, HDL, GAD, HbA1c, FCP, P2hCP, HOMA-β and use of insulin were statistically different between two groups. The levels of BMI, TG, LDL, FCP, P2hCP and HOMA-β in the LADA-2 group were higher than those in the LADA-1 group, while the levels of GAD, HbA1c and and use of insulin were lower than the LADA-1 group. There was no significant difference in the proportion of male, age, duration, SBP, DBP, TC, HDL, ALT, AST, Cr SUA and HOMA-IR.

**Table 2:** Clinical characteristics and islet function indexes of total subjects according to GAD values

	LADA-1	LADA-2	P
GAD(IU/ml)	≥ 180	< 180	
n	136	351	
male (%)	66(48.5%)	164(46.7%)	0.139
Age (years)	58.8 ± 16.3	58.1 ± 15.2	0.741
duration(years)	6.7 ± 4.4	7.0 ± 4.3	0.115
BMI (kg/m^2^)	19.4 ± 4.3	23.6 ± 4.2	0.034
SBP (mmHg)	127.8 ± 17.3	128.9 ± 18.7	0.259
DBP (mmHg)	70.5 ± 16.7	71.2 ± 18.6	0.395
TG (mmol/L)	1.4 ± 0.7	1.6 ± 0.6	0.017
TC (mmol/L)	4.3 ± 0.8	4.4 ± 0.9	0.085
LDL (mmol/L)	2.6 ± 0.6	2.7 ± 0.7	0.033
HDL (mmol/L)	1.0 ± 0.4	1.0 ± 0.5	0.096
GAD(IU/ml)	272.0 ± 62.7	57.2 ± 54.3	< 0.001
ALT (U/L)	19.7 ± 12.7	21.2 ± 13.6	0.329
AST (U/L)	19.8 ± 13.1	20.5 ± 12.8	0.413
Cr (umol/L)	64.9 ± 30.2	63.5 ± 29.7	0.154
SUA (umol/L)	324.2 ± 145.8	323.7 ± 151.3	0.469
HbA1c(%)	9.4 ± 2.4	9.1 ± 2.1	0.024
FCP (ng/ml)	1.0 ± 0.7	1.7 ± 0.7	0.013
P2hCP (ng/ml)	1.7 ± 0.8	2.4 ± 0.8	0.012
Use of insulin(%)	88(64.7%)	136(38.7%)	< 0.001
HOMA-IR	1.6 ± 0.6	1.7 ± 0.6	0.079
HOMA-β	11.0 ± 5.7	14.7 ± 6.3	0.019

Values are mean ± SD.

Tables 3 and 4 showed the distributions of clinical characteristics and islet function indexes of the patients according to GAD-specific tertiles of RDW levels, respectively. In LADA-2 group, participants with a higher RDW were significantly more likely to be older, female sex, a longer duration of diabetes and higher levels of TG and Cr. The analysis of islet function indexes showed there were significant difference in FCP, P2hCP and HOMA-β among the three tertiles; however, it did not show a slightly increasing trend in HOMA-IR. In LADA-1 group, there was significant statistical difference with respect to age, duration, Cr and FCP levels among the subgroups while all other variables did not differ significantly. Specifically, paired comparisons showed significant difference between tertile 1 and tertile 3 of HOMA-β values in LADA-1 group after Bonferroni correction, but no statistical significance was demonstrated between tertile 1 and tertile 2 or between tertile 2 and tertile 3.

**Table 3:** Clinical characteristics and islet function indexes of LADA-2 subjects according to RDW tertiles

	I(11.2–12.4%)	II(12.5–12.9%)	III(13.0–18%)	P
n	117	118	116	
male (%)	69(59.0%) ^#Δ^	53(44.9%)^*Δ^	42(36.2%)^*#^	< 0.001
Age (years)	56.8 ± 13.9^#Δ^	58.2 ± 14.5^*Δ^	59.1 ± 14.2^*#^	< 0.001
duration(years)	6.6 ± 4.1^#Δ^	7.0 ± 4.2^*Δ^	7.3 ± 4.1^*#^	0.018
BMI (kg/m2)	22.9 ± 3.9	23.9 ± 4.5	23.8 ± 4.0	0.074
SBP (mmHg)	128.3 ± 18.1	129.4 ± 17.9	129.1 ± 18.5	0.317
DBP (mmHg)	70.9 ± 17.8	71.4 ± 17.9	71.2 ± 17.3	0.562
TG (mmol/L)	1.8 ± 0.5^#Δ^	1.6 ± 0.5^*Δ^	1.4 ± 0.6^*#^	0.021
TC (mmol/L)	4.3 ± 0.6	4.5 ± 0.7	4.4 ± 0.8	0.263
LDL (mmol/L)	2.7 ± 0.6	2.8 ± 0.6	2.8 ± 0.6	0.094
HDL (mmol/L)	1.0 ± 0.4	1.0 ± 0.4	1.0 ± 0.3	0.173
GAD(IU/ml)	56.7 ± 52.1	54.8 ± 42.9	59.9 ± 51.6	0.316
ALT (U/L)	19.4 ± 12.8	21.8 ± 13.2	22.4 ± 13.0	0.219
AST (U/L)	19.7 ± 12.1	21.8 ± 11.9	20.1 ± 12.4	0.594
Cr (umol/L)	60.7 ± 28.9^#Δ^	63.1 ± 29.1^*Δ^	66.7 ± 29.2^*#^	0.038
SUA (umol/L)	318.9 ± 146.7	331.1 ± 150.6	321.2 ± 149.9	0.297
HbA1c(%)	9.1 ± 1.9	9.1 ± 2.0	9.0 ± 2.1	0.062
FCP (ng/ml)	1.6 ± 0.6^#Δ^	1.7 ± 0.6^*Δ^	1.8 ± 0.7^*#^	0.021
P2hCP (ng/ml)	1.9 ± 0.7^#Δ^	2.4 ± 0.7^*Δ^	2.8 ± 0.7^*#^	< 0.001
Use of insulin(%)	47(40.2%)	45(38.1%)	44(38.0%)	0.197
HOMA-IR	1.6 ± 0.5	1.7 ± 0.6	1.6 ± 0.6	0.315
HOMA-β	12.7 ± 5.8^#Δ^	14.8 ± 5.9^*Δ^	16.7 ± 6.1^*#^	< 0.001

Values are mean ± SD,*P < 0.05 VS tertile 1; # P < 0.05 VS tertile 2;ΔP < 0.05 VS tertile 3

**Table 4:** Clinical characteristics and islet function indexes of LADA-1 subjects according to RDW tertiles

	I(11.2–12.4%)	II(12.5–12.9%)	III(13.0–18%)	P
n	46	46	44	
male (%)	24	22	20	0.323
Age (years)	57.3 ± 15.6#Δ	58.8 ± 16.8*Δ	61.2 ± 16.4*#	0.037
duration(years)	5.8 ± 3.5#Δ	6.8 ± 3.6*Δ	7.0 ± 3.8*#	0.027
BMI (kg/m2)	19.1 ± 3.4	19.6 ± 3.5	19.5 ± 3.6	0.676
SBP (mmHg)	126.9 ± 16.5	128.9 ± 15.9	127.9 ± 16.7	0.351
DBP (mmHg)	69.1 ± 16.3	70.9 ± 15.8	71.4 ± 16.5	0.092
TG (mmol/L)	1.2 ± 0.5	1.4 ± 0.6	1.4 ± 0.6	0.479
TC (mmol/L)	4.34 ± 0.74	4.02 ± 0.59	4.46 ± 0.76	0.294
LDL (mmol/L)	2.6 ± 0.4	2.4 ± 0.5	2.8 ± 0.6	0.531
HDL (mmol/L)	1.1 ± 0.3	1.0 ± 0.3	0.9 ± 0.4	0.072
GAD(IU/ml)	278.4 ± 54.2	273.0 ± 57.4	264.5 ± 55.9	0.095
ALT (U/L)	18.8 ± 11.9	21.1 ± 11.7	19.1 ± 10.9	0.413
AST (U/L)	18.7 ± 12.1	21.4 ± 12.9	19.3 ± 11.8	0.349
Cr (umol/L)	63.5 ± 29.3#Δ	64.8 ± 28.7*Δ	66.3 ± 29.6*#	0.044
SUA (umol/L)	318.8 ± 140.2	329.6 ± 139.7	324.2 ± 141.8	0.278
HbA1c(%)	9.5 ± 2.2	9.4 ± 2.3	9.3 ± 2.3	0.072
FCP (ng/ml)	0.8 ± 0.4#Δ	0.9 ± 0.5*Δ	1.1 ± 0.7*#	< 0.001
P2hCP (ng/ml)	1.6 ± 0.5	1.8 ± 0.6	1.8 ± 0.6	0.064
Use of insulin(%)	29(63.0%)	30(65.2%)	29(65.9%)	0.164
HOMA-IR	1.6 ± 0.5	1.5 ± 0.5	1.6 ± 0.5	0.315
HOMA-β	10.4 ± 5.2Δ	11.19 ± 4.9	11.29 ± 5.2*	0.082

Values are mean ± SD,*P < 0.05 VS tertile 1; # P < 0.05 VS tertile 2;ΔP < 0.05 VS tertile 3

The correlation between RDW and clinical characteristics and islet function indexes were shown in Table 5. In the total population, correlation analysis revealed that RDW significantly correlated with male sex, age, duration, TG, Cr, FCP, and HOMA-β. In LADA-1 subjects, RDW correlated positively with age, duration, Cr, HbA1c and FCP. In LADA-2 subjects, RDW correlated positively with age, duration, Cr, FCP, P2hCP and HOMA-β, and it correlated negatively with male sex, TG.

**Table 5:** correlation of selected variables with RDW in LADA patients in total and subgroups

	total		LADA-1		LADA-2	
	r	P	r	P	r	P
male (%)	-0.215	< 0.001	-0.042	0.136	-0.267	< 0.001
Age (years)	0.097	0.014	0.052	0.011	0.074	0.007
duration(years)	0.183	0.006	0.089	0.022	0.142	0.003
TG (mmol/L)	-0.132	0.008	-0.057	0.064	-0.106	0.019
Cr (umol/L)	0.087	0.042	0.048	0.032	0.042	0.035
HbA1c(%)	-0.078	0.065	-0.198	0.007	-0.053	0.097
FCP (ng/ml)	0.092	0.032	0.058	0.037	0.162	0.014
P2hCP (ng/ml)	0.053	0.136	0.073	0.087	0.152	0.042
HOMA-β	0.118	0.027	0.148	0.076	0.218	0.009
HOMA-IR	-0.013	0.432	-0.012	0.317	0.009	0.218

To examine the influence of independent variables on the dependent variable of RDW, multiple linear regression analyses were performed using RDW as the dependent variable (Table 6). In unadjusted analyses, the relation between RDW and HOMA-β were statistically significant in total population and LADA-2 patients, but not in LADA-1 patients. After adjusted potentially important factors,(mode 2: age, BMI, sex and diabetes duration; Model3: adjusted for age, BMI, sex, diabetes duration and HbA1c), RDW remained positively associated with HOMA-β in LADA-2 patients.

**Table 6:** Multiple linear regression analysis for RDW and HOMA-β in LADA patients in total and subgroups

		B	SE	β	t	P
Total	RDW (Model 1)	0.012	0.003	0.152	3.542	0.007
	RDW (Model 2)	0.012	0.003	0.137	2.985	0.009
	RDW (Model 3)	0.012	0.003	0.115	2.047	0.032
LADA-1	RDW (Model 1)	0.007	0.001	0.098	1.524	0.069
	RDW (Model 2)	0.007	0.001	0.087	1.412	0.078
	RDW (Model 3)	0.007	0.001	0.065	1.291	0.103
LADA-2	RDW (Model 1)	0.016	0.004	0.204	3.774	0.006
	RDW (Model 2)	0.016	0.004	0.186	3.042	0.009
	RDW (Model 3)	0.016	0.004	0.173	2.654	0.017

Model1: unadjusted; Model 2: adjusted for age, BMI, sex and diabetes duration; Model3: adjusted for age, BMI, sex, diabetes duration and HbA1c;

Multiple regression analysis in Table 7 were carried out using RDW as the dependent variable to explore the association between RDW and HbA1c. After adjustment for the variables confounders (age, BMI, sex, diabetes duration), RDW were significantly associated with HbA1c in LADA-1 patients, but the correlation was not found in LADA-2 patients in either unadjusted analyses or adjusted analysis.

**Table 7:** Multiple linear regression analysis for RDW and HbA1c in LADA patients in total and subgroups

		B	SE	β	t	P
Total	RDW (Model 1)	-0.067	0.019	-0.124	-1.982	0.052
	RDW (Model 2)	-0.047	0.011	-0.097	-2.737	0.067
LADA-1	RDW (Model 1)	-0.120	0.037	-0.285	-2.297	0.001
	RDW (Model 2)	-0.098	0.027	-0.139	-2.196	0.002
LADA-2	RDW (Model 1)	-0.034	0.009	-0.096	-1.673	0.084
	RDW (Model 2)	-0.029	0.008	-0.075	-1.428	0.120

## Discussion

Our study is, to the best of our knowledge, the first to investigate the association between RDW and islet β-cell function indexes in patients of LADA. We demonstrate that RDW is associated with beta cell function in LADA individuals with low GAD titers. The association persisted after adjustment for potential confounding factors. However, there was a lack of correlation between RDW and HOMA-IR in LADA patients.

Among diabetic patients, chronic hyperglycemia will lead to non-enzymatic glycation RBC membrane proteins that would accelerate RBC aging. During erythropoiesis, RBC structure or chemistry will be affected by hyperglycemic state and these changes could have an imposing effect on red cell indices, which include the red cell shape and deformability represented by RDW [11, 12]. Previous studies have found a positive correlation of RDW and HOMA-β among type 2 diabetes, which is similar to our study that the relationship is found among LADA patients with low GAD titer [13]. Controversy exists regarding whether LADA is an intermediate phenotype between typical type1 and type 2 diabetes or obese type 1 diabetes. In our research, we only found that RDW levels were significantly associated with HOMA-β in LADA patients with low GAD titers, but not in patients with high GAD titers. This phenomenon may be explained that those LADA patients who have low titers of a single autoantibody have clinical, biochemical, and genetic characteristics more similar to type 2 diabetes, which features chronic low-grade inflammation distinct from type 1 diabetes [14–16]. Chronic inflammatory process related to hyperglycemia may influence erythropoiesis and would reduce RBCs half-life and deformability, thereby increasing RDW [12].

It is well known that insulin is a general regulator of protein synthesis and it exerts a growth promoting activity in various kinds of cells. An increment in the level of insulin could be leading to an increase in red blood cell synthesis. In our study, C-peptide as a marker of islet secretion function, is positively correlated with RDW. Previous studies demonstrate in vitro that insulin stimulates the proliferation of erythroid progenitors, and it may thus play a stimulatory role in human erythropoiesis [17, 18]. In vivo and vitro experiments, experts find that erythropoietin significantly decreased blood glucose level [19]. Therefore, it might be speculated that innate increasing of beta-cell secretion, may be an underlying factor for erythrocyte metabolism. However, unlike our study, Salami et al. found decreased levels of RDW were associated with increased fasting insulin among islet autoantibody positive children with increasing risk for type 1 diabetes [9]. On the other hand, some of the patients in our study were treated with exogenous insulin which might affect erythropoiesis. To our knowledge, there are few reports about the effects of exogenous insulin on RDW.AM Nada observed no significant effects of the hypoglycemic agents including insulin on RDW[20]. Xu et al. found that after short-term continuous subcutaneous insulin infusion (CSII), patients with lower baseline RDW are more likely to maintain a one-year euglycemia remission[21].

Several studies have investigated the association between RDW and HbA1c among heterogenous populations, and the observations are variable. Some researchers have reported that RDW has a significant positive correlation with HbA1c[22]. Other studies demonstrated diabetes subjects with higher RDW had substantially lower risk of being in poor glycemic control [23]. Veeranna et al. found RDW was independently associated with HbA1c among 15,343 nondiabetic adults raising the possibility of chronic hyperglycemia along with oxidative stress and inflammation as a mediating link between RDW and its association with cardiovascular outcomes [24]. Peterson et al. observed a modest but consistent increase in erythrocyte half-life after the establishment of tight glycemic control compared with the same patients studied in poor control.AM Nada showed good glycemic control was associated with lower RDW than in patients with poor control. There was a significant difference in RDW, being significantly higher in patients with HbA1c > 7%, indicating shorter life span with anisocytosis in uncontrolled diabetes. However, Cakir et al. did not find a significant difference in RDW in patients with HbA1c < 7% or > 7%.

Comparing with the previous studies, we observed a negative correlation between RDW and HbA1c in LADA patients with high GAD titers but not with low GAD titers. In recent years LADA patients are supposed to be more consistent with a heterogeneous population of type 1 and type 2 diabetes rather than a single intermediated phenotype [25]. LADA patients with high GAD titers will be more like patients with type 1 diabetes-for example, to be younger at diagnosis, have decreased BMI, and be more likely to progress to insulin [26–29]. Similar to our finding, the decrease in RBC indices with increasing HbA1c were observed in islet autoantibody positive children with increased genetic risk for type 1 diabetes, and the study followed for 5 years [9]. The significant negative association suggests the mechanism may originate from early haematopoiesis in the bone marrow. The decrease of red blood cell counts and RDW, levels of haemoglobin with the increased level of glucose are all indicating a disorder of the red cell homeostasis and function associated with impaired glucose metabolism. The correlation between RDW and HbA1c may be the consequence of a progressive increase of frailty of red blood cells, which contributing to chronic modifications induced by glucose on the morphological and hemorheological characteristics of erythrocytes [30].

However, our study has several limitations. First, the cross-sectional design limited the investigation of the progress of beta cell function. Second, since the patients recruited were inpatients with poor blood glucose control, a selection bias could be introduced in our study. Third, only data for RDW from the first 24 h of admission were selected. Thus, the association between subsequent changes in RDW was not evaluated. We used baseline assessment only, which could increase the risk of misclassification bias. It is worth mentioning that the value of modified HOMA-β (based on fasting glucose and C-peptide levels) were not validated versus clamp assessments. Further scientific work is warranted to confirm our results. The information on exogenous insulin replacement were inexhaustive, which is intrinsically related to RDW and may affect our results. Nevertheless, this is the first reported study to determine the relationship between RDW and beta cell function in LADA patients. Therefore, more accurate and generalized results might be obtained by performing a cohort study.

## Conclusion

In conclusion, RDW was positively associated with beta cell function in LADA individuals with low GAD titers. We observed a negative correlation between RDW and HbA1c in LADA patients with high GAD titers but not with low GAD titers. However, we need further large-scale studies or cohort studies to verify the relationship and interaction mechanism between RDW and beta cell function among LADA patients.

## Data Availability

The datasets used and analysed during the current study are available form the corresponding author on reasonable request.

## Abbreviations

LADA: Latent autoimmune diabetes in adults
RDW: Red blood cell distribution width
HbA1c: Glycosylated hemoglobin A1c
HOMA-IR: Homeostasis model assessment of insulin resistance
HOMA-β: Homeostasis model assessment of β-cell function
HOMA: Homeostasis model assessment
FBG: Fasting blood glucose
FCP: Fasting C-peptide index
P2hCP: Postprandial 2 h C-peptide
TG: Triglycerides
TC: Total cholesterol
LDL: Low-density lipoprotein cholesterol
HDL-C: High-density lipoprotein cholesterol
Cr: Creatinine ALT:glutamic-pyruvic transaminase
AST: Glutamic oxalacetic transaminase
RBC: Red blood cell
MCV: Mean corpuscular volume
COVID-19: Coronavirus disease 2019
T2DM: Type 2 diabetes mellitus
WHO: World Health Organization
SBP: Systolic blood pressure
DBP: Diastolic blood pressure
BMI: Body mass index
SUA: Serum uric acid
SPSS: Statistical Product and Service Solutions
SD: Standard deviations
ANOVA: The one-way analysis of variance
CBC: Complete blood count
WC: Waist circumference
